# Supplementary material for: Saccharomyces boulardii (CNCM I-745) improves intestinal damage in sepsis by remodeling intestinal flora structure
Source: Front Cell Infect Microbiol. 2025 Jul 31;15:1602792. doi: 10.3389/fcimb.2025.1602792 (PMC12350329; doi:10.3389/fcimb.2025.1602792)
Supplement: Supplementary file 1 [file DataSheet1.zip › summary/summary/8_Advanced_analyse/2_PICRUSt2_result/KEGG/Group.CW4_vs_AW4/PICRUSt2_KEGG_level3_Group.CW4_vs_AW4_diff.pdf]

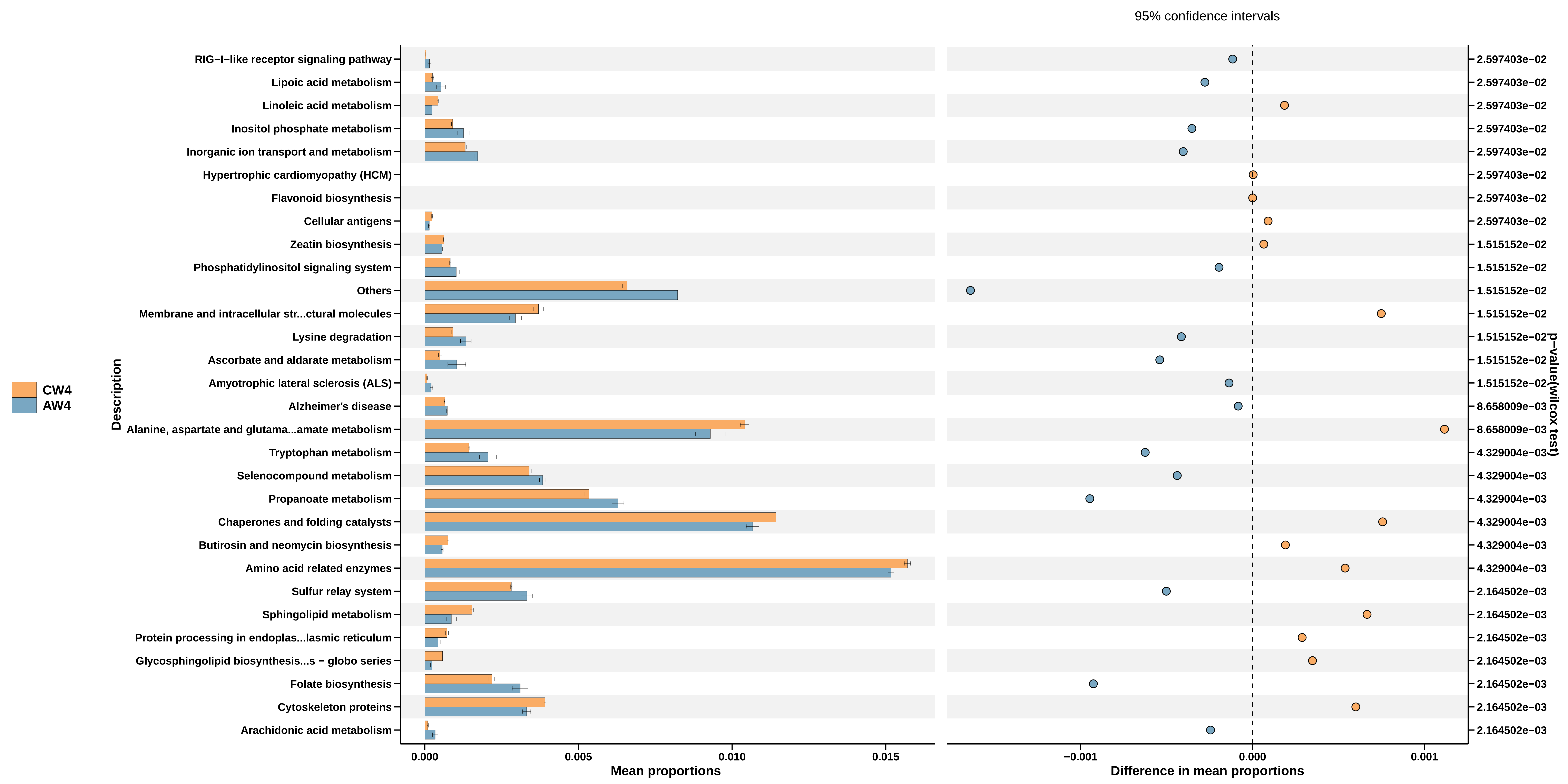

95% confidence intervals
CW4
AW4
Description
RIG−I−like receptor signaling pathway
Lipoic acid metabolism
Linoleic acid metabolism
Inositol phosphate metabolism
Inorganic ion transport and metabolism
Hypertrophic cardiomyopathy (HCM)
Flavonoid biosynthesis
Cellular antigens
Zeatin biosynthesis
Phosphatidylinositol signaling system
Others
Membrane and intracellular str...ctural molecules
Lysine degradation
Ascorbate and aldarate metabolism
Amyotrophic lateral sclerosis (ALS)
Alzheimer's disease
Alanine, aspartate and glutama...amate metabolism
Tryptophan metabolism
Selenocompound metabolism
Propanoate metabolism
Chaperones and folding catalysts
Butirosin and neomycin biosynthesis
Amino acid related enzymes
Sulfur relay system
Sphingolipid metabolism
Protein processing in endoplas...lasmic reticulum
Glycosphingolipid biosynthesis...s − globo series
Folate biosynthesis
Cytoskeleton proteins
Arachidonic acid metabolism
0.000
0.005
0.010
0.015
Mean proportions
−0.001
0.000
0.001
Difference in mean proportions
p−value(wilcox test)
2.597403e−02
2.597403e−02
2.597403e−02
2.597403e−02
2.597403e−02
2.597403e−02
2.597403e−02
2.597403e−02
1.515152e−02
1.515152e−02
1.515152e−02
1.515152e−02
1.515152e−02
1.515152e−02
1.515152e−02
8.658009e−03
8.658009e−03
4.329004e−03
4.329004e−03
4.329004e−03
4.329004e−03
4.329004e−03
4.329004e−03
2.164502e−03
2.164502e−03
2.164502e−03
2.164502e−03
2.164502e−03
2.164502e−03
2.164502e−03
